# Influence of Redispersible Powder on Properties of Self-Leveling Mortar of Ternary Cementitious System

**DOI:** 10.3390/ma13245703

**Published:** 2020-12-14

**Authors:** Wenyan Dong, Congqi Fang, Ran Hu

**Affiliations:** 1 Department of Civil Engineering, Shanghai Jiao Tong University, Shanghai 200240, China; dwy921@sjtu.edu.cn (W.D.); m17839999168@163.com (R.H.); 2 School of Civil Engineering, Shanghai Normal University, Shanghai 201418, China

**Keywords:** self-leveling mortar, redispersible powder, ternary cementitious system, analytic hierarchy process

## Abstract

The self-leveling mortar (SLM) of a ternary cementitious system with different dosages of redispersible powder (RP) with ordinary Portland cement (OPC), sulfoaluminate cement (SAC), and calcium sulfate (CS) as cementitious materials was investigated with regard to fluidity, bond strength, shrinkage rate, abrasion resistance, flexural strength, and compressive strength. The performance parameters obtained from the experimental test for SLM were weighted values calculated with an analytic hierarchy process (AHP). The comprehensive index of performance was evaluated on the basis of a weighted-sum method, and the optimal dosage of RP was determined according to the comprehensive index. The experimental results demonstrated that the fluidity of SLM decreased with the increase in RP dosage at the beginning but then increased thereafter and decreased rapidly as the dosage went beyond 3.0%. The addition of RP resulted in a significant improvement in bond strength (of SLM), reduction in the shrinkage rate, abrasion loss, early flexural strength and compressive strength, and resistance to cracking. The properties of SLM with 3.0% RP can meet the requirements of the industrial standard for cementitious self-leveling floor mortar. Compared with the SLM without RP, the bond strength of SLM with 3.0% RP was increased by 46.7%, while the shrinkage rate and abrasion loss were reduced by 50% and 71.9% respectively. The weighted values of fluidity, compressive strength, flexural strength, stability, cohesiveness, and abrasion resistance were 0.422, 0.196, 0.196, 0.089, 0.058, and 0.039, respectively. A higher value of the comprehensive index generally denotes a better performance. The comprehensive index of SLM with 3.0% RP was the highest.

## 1. Introduction

Self-leveling mortar (SLM) used as a floor material consist of cements, fine aggregates, mineral fillers, and chemical admixtures. When mixed with water, it can be leveled with high fluidity or a little auxiliary paving. Owing to the advantages of high fluidity, simple construction, good flatness, high compressive strength, and thin leveling layer, it is an ideal ground material for constructions in large supermarkets, shopping malls, parking lots, factory workshops, and warehouses [1]. Conventional SLM usually uses Portland cement (PC) as a cementitious material. However, due to the low early strength and serious drying shrinkage of PC [2], cracks and curls commonly appear at the corners and perimeter. Therefore, it is difficult for conventional SLM to meet the requirements of early strength and crack resistance if only PC is used. In order to achieve better performance, SLM with binary or ternary cementitious systems came into being. Binary or ternary cementitious systems refer to composites of PC, calcium aluminate cement (CAC) or sulfoaluminate cement (SAC), and calcium sulfate (CS).

For SLM with a binary cementitious system, a series of studies were carried out. Zhang et al. [3] studied the influence of CAC on the performance of SLM (binary cementitious system: PC–CAC), and obtained the optimum dosage of CAC. By studying the properties of SLM (PC–SAC), Georgin et al. [2], Pera et al. [4], and Wang et al. [5] found that SAC could reduce the dry shrinkage of SLM, as well as improve the flexural and compressive strength of mortar. The properties of sulfoaluminate cement-based SLM with CS were studied by Li et al. [6,7], who obtained that the formation of ettringite in the early term mainly contributes to strength development, while the formation of ettringite at a later hydration stage favors a decrease in late-term drying shrinkage.

With the development of research on SLM, scholars also carried out many researches on SLM with ternary cementitious systems [8,9,10,11,12,13,14,15]. SLM composed of CAC, ordinary PC (OPC), and CS had better hardening characteristics, faster drying, and better dimensional stability [8,9]. Zhang et al. [10] studied the effect of CS type and dosage on the properties of SLM. The test results revealed that, compared to other CS, SLM prepared with anhydrite exhibited the highest initial flow value and setting time and the lowest fluidity loss. The dihydrate was favorable for the early strength. Mortar containing α-hemihydrate exhibited more expansion. The shrinkage compensating effect became more obvious with the increase in CS in SLM samples. According to research on the effect of curing temperature on the hydration of CAC–PC–CS blends [11,12], it was found that, compared to anhydrite, hemihydrate is more suitable to be applied in conditions of high temperature (40 °C). The study by David et al. [13] found that the reaction mechanism of a ternary cementitious system (PC–CAC–CS) depends on the CAC and CS amount in the binders. Laure et al. [14] studied the hydration mechanism and properties of mortar with a ternary system (OPC–CAC–CS). The results showed CSA clinker was mainly responsible for the early mechanical properties, while OPC played an important role at the later stages. Through the study of mechanical properties of SLM composed of a OPC–SAC–PG (phosphogypsum) ternary cementitious system [15], it was found that the increase in compressive and flexural strength of mortar was not affected by PG, but the shrinkage, bond strength, and wear resistance were greatly affected.

It can be seen that cementitious materials are the important influencing factors for the performance of SLM. On the other hand, as an important component of SLM, the chemical admixtures (water reducer, retarder, early strength agent, and stabilizer) in the mortar also affect the performance of SLM [8,16,17,18,19,20,21]. For example, the retarder can prolong the setting time and increase the fluidity of SLM. With an increase in retarder content, the strength of SLM increases first and then decreases.

RP is prepared by spray-drying or other process of polymer emulsion. When the powder mixes with water, the properties of the polymer emulsion are similar to those of the original emulsion [22,23]. As an important chemical admixture, although the amount of RP is very small, it has a great influence on the performance of mortar. Do [24] found that the polymer modified PC-based SLM can be compared with the traditional floor facing material of thermosetting resin in practical applications. Sun et al. [25] studied the influence of different types of RP on the performance of SLM. The results showed that RP can significantly improve the fluidity and wear resistance of self-leveling mortar. On the other hand, RP reduces the compressive strength of mortar, whereas it improves the flexural strength, the bond strength, the impact resistance, and the flexibility of mortar [26,27,28,29,30,31]. In addition, Schulze et al. [23] also studied the long-term performance of the RP-modified PC mortar and concluded that, after 10 years, the microstructure of the polymer in the mortar still did not change, maintaining a stable bond, flexural, and compressive strength.

Therefore, RP is an indispensable additive component for excellent mortar as a floor material. However, if the dosage of RP is not in the appropriate range, it will increase the cost and be harmful to the performance of mortar [16]. Therefore, it is valuable to conduct studies on the effect of its dosage on the performance of SLM. The existing researches primarily focused on the performance of the RP in SLM with binary systems. Few researches dealt with the influence of RP on the performance of SLM with ternary cementitious systems (OPC–SAC–CS). As the mortar is a complex system composed of a variety of materials, the requirements of material type and dosage are very strict. If the material type or dosage is slightly different, the performance will vary significantly.

Although there were many researches on the different kinds of SLM, the researches on SLM with ternary cementitious systems composed of OPC–SAC–CS are still limited, especially the effect of additives on its performance. As an important additive that affects the properties of materials, in order to obtain a better SLM with ternary cementitious system, it is necessary to study the effect of RP on the properties of mortar and determine a reasonable dosage. The current study experimentally investigated the influence of RP on the performance of SLM with a ternary cementitious system (OPC–SAC–CS). The investigation was conducted by testing the fluidity, bond strength, shrinkage rate, abrasion resistance, flexural strength, and compressive strength. How to build a complete and unified mortar performance evaluation method on the basis of the properties and data measured in the test is the key to determining the reasonable RP dosage. This paper introduced a method to determine the reasonable dosage of RP through an analytic hierarchy process (AHP). In this method, a hierarchy structure model for performance evaluation of mortar based on tests was first established, then the weighted values of fluidity, mechanical properties, and other evaluation indices were determined, and finally the comprehensive performance evaluation indices of mortar were calculated to determine the reasonable dosage of RP.

## 2. Materials and Methods

### 2.1. Materials

**Figure 1:**
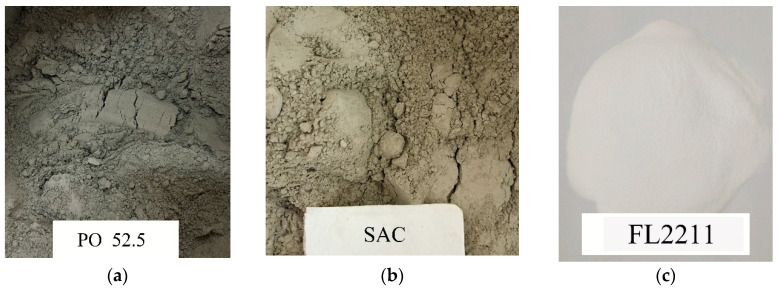
Materials of self-leveling mortar (SLM): (**a**) ordinary Portland cement (OPC); (**b**) sulfoaluminate cement (SAC); (**c**) redispersible powder (RP).

**Figure 2:**
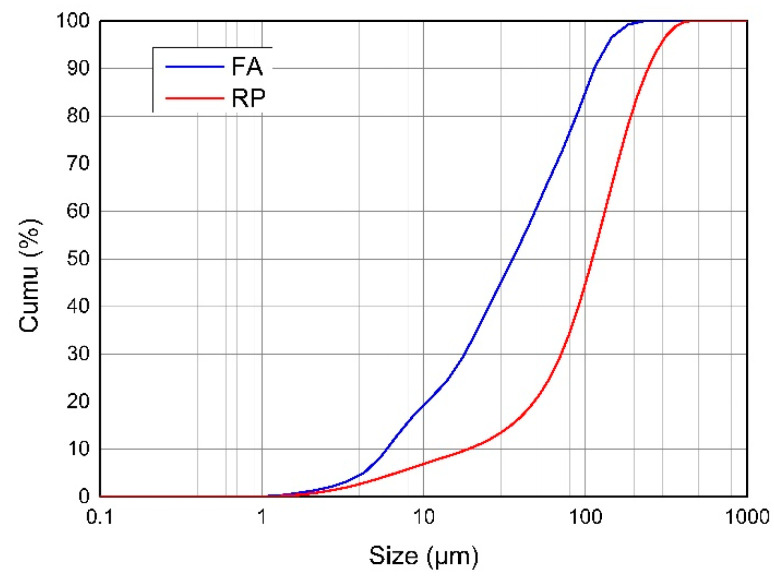
Particle size distribution of fly ash (FA) and RP.

OPC (type PO 52.5) and SAC, as shown in Figure 1a, b, were obtained from Shanghai Hailuo Cement Co., Ltd. (Shanghai, China) and Tangshan Polar Bear Building Materials Co., Ltd. (Tangshan, China) respectively. The chemical composition of the cements was determined by X-ray fluorescence (XRF) (Shimadzu (China) Co., Ltd.) (Shanghai, China), as shown in Table 1. The specific surface areas of OPC and SAC were 374 m^2^/kg and 455 m^2^/kg, respectively. The CS used in this research included anhydrite and α-hemihydrate. The fine aggregates were composed of coarse quartz sand (16–40 mesh) and fine quartz sand (30–100 mesh). Fly ash (FA) and ground calcium carbonate (GCC) were used as mineral fillers. The Blaine fineness of GCC was 325 mesh. The redispersible powder (RP, Elotex FL2211) was obtained from Akzonobel (Shanghai, China), as shown in Figure 1c. Its basic properties are listed in Table 2. FL2211 is redispersible vinyl acetate and ethylene copolymer power. The particle size distributions of FA and RP are shown in Figure 2. The other chemical admixtures used in this research included polycarboxylate-based superplasticizer (SP) dry powder, UEA expansive agent, P803 antifoaming agent, and MT400 cellulose ether.

### 2.2. Mix Proportion

In total, seven formulations with different redispersible powder dosages (0%, 0.5%, 1.0%, 1.5%, 2.0%, 3.0%, and 4.0%) were used (R0, R0.5, R1, R1.5, R2, R3, and R4, respectively). The details of SLM mix formulations is given in Table 3 in which all the components were calculated by mass percentage.

The amounts of both anhydrite and α-hemihydrate were 50% of the CS. The dosages of coarse quartz sand and fine quartz sand were 214.5 kg/m^3^ and 212.5 kg/m^3^, respectively. Admixtures 1 denotes the chemical admixtures expected for redispersible powder. The dosages of polycarboxylate-based superplasticizer, expansive agent, antifoaming agent, and cellulose ether were 2.9 kg/m^3^, 29 kg/m^3^, 1 kg/m^3^, and 0.5 kg/m^3^, respectively. The rate of water to cementitious materials was 0.24.

### 2.3. Test Sample Prepartion

According to standard JC/T 985-2005 [32] (Chinese standard for cementitious self-leveling floor mortar), all the samples were prepared in the standard experimental conditions of 23 ± 2 °C and 50% ± 5% relative humidity. All materials were stored in the standard experimental conditions for at least 24 h before mixing.

All samples were prepared following the procedures proposed in JC/T 985–2005 [32]. The details of mixing procedure were as follows: according to the above mix formulations, all materials (except water) were firstly dry-mixed to be homogeneous. The corresponding amount of water was mixed into the blender. The premixed materials were added to the water at a same speed within 30 s and then the mixtures were mixed at slow speed for 1 min. Thereafter, the mixtures were mixed at higher speed for 1 min. Then, a holding period of 5 min was applied, followed by 15 s of higher-speed mixing. Finally, the mortars were casted if there were no air bubbles. Otherwise, they were held for another minute before casting.

The fluidity of mortars was measured immediately after mixing. For other tests, mortar was poured into molds of different sizes and cured in standard experimental conditions. The samples were demolded after 24 h. After demolding, the samples were cured to different ages in standard experimental conditions to test the corresponding performance.

### 2.4. Test Methods

#### 2.4.1. Fluidity

Fluidity was measured according to JC/T 985-2005 [32]. It was measured twice to indicate initial fluidity and 20 min fluidity. The initial fluidity was tested as soon as the mixing procedure was completed, and the 20 min fluidity was tested after holding the same batch for 20 min. The fluidity test mold with an inner diameter of 30 mm ± 0.1 mm and a height of 50 mm ± 0.1 mm was placed horizontally at the center of the plate glass. The mold was filled with fresh mortar and raised vertically within 2 s to allow the mortar to flow freely. After 4 min, the diameters of mortar in two orthogonal directions were measured and the average value was noted as the initial fluidity of mortar. A sample of the same batch was placed in the blender for 20 min, and the diameter was measured in the same way as above. The diameter was recorded as the fluidity of mortar after 20 min. For either initial or 20 min fluidity, the test was carried out twice and the average was taken as the final result with an accuracy of 1 mm.

#### 2.4.2. Bond Strength

For the bond strength, 10 SLM samples of each mix proportion sized 50 mm × 50 mm × 5 mm were molded on a concrete board using a silicone rubber frame and were cured in standard conditions for 28 days. Then, the steel drawing heads were bonded to the samples using epoxy resin. After hardening, the bond strength between the SLM and concrete board was measured using an electronic tensile testing machine. The bond strength was given by Equation (1).

*P* = F/S (1)

where *P* is the bond strength (MPa), F is the largest fracture load (N), and S is the bond area (2500 mm^2^).

Test results were accurate to 0.1 MPa. The average of 10 values was calculated, and those values outside the range of ±20% of the average were discarded. At least five values for each mix proportion were retained to ensure the reproducibility of bond strength tests. If five or more values were retained, a new average was calculated. If the number of available values was less than five, the test was repeated. The average of effective results was determined as the final bond strength. If a break took place between the steel drawing head and the sample, this result was discarded and the test was repeated.

#### 2.4.3. Shrinkage Rate

For the shrinkage rate, three SLM samples (40 mm × 40 mm × 160 mm) with copper probes embedded were molded without vibrating for each mix proportion. The samples were demolded after 24 h of curing in standard conditions, and the initial lengths were immediately measured using a mortar shrinkage dilatometer. Then, the samples continued to be cured at the same environment for 28 days and the lengths were measured again. The change in the length of samples was recorded, and the shrinkage rate was calculated using Equation (2).

(2) $$\varepsilon =\frac{L_{t}-L_{0}}{L-L_{d}}\times 100\%$$

where  ε  is the shrinkage rate (%), *L*_0_ is the initial length of sample (mm), *L_t_* is the length at 28 days (mm), *L* = 160 mm, and *L_d_* is the total depth of the embedded copper probes (20 mm). The average value of three samples was defined as the final shrinkage rate.

#### 2.4.4. Abrasion Resistance

For the abrasion resistance, two of the SLM samples were molded using a metal mold (with an inner diameter of 105 mm and a height of 5 mm) without vibrating for each mix proportion. The samples were demolded after 24 h of curing in standard conditions. Then, the samples continued to be cured in the same environment for 28 days. The test of abrasion resistance was carried out using an abrasion instrument. The turntable speed was 60 ± 2 rpm and load weight was 500 g. First, the surface of the samples was wiped, and the weights of the samples were recorded. Then, the samples were abraded with an abrasion machine. The surface ash was cleaned after the abrading was completed. Weights of the samples were recorded as the weights of the samples after grinding. The abrasion loss was calculated using Equation (3).

F = G_0_ − G_1_ (3)

where F is the abrasion loss (g), G_0_ the initial weight of sample before abrading (g), and G_1_ is the weight of the sample after abrading (g). The abrasion resistance is described by the average abrasion loss of two samples.

#### 2.4.5. Flexural Strength and Compressive Strength

For flexural strength and compressive strength, SLM samples with a size of 40 mm × 40 mm × 160 mm were casted using metal molds. For each mix proportion, three groups of SLM samples were prepared for the determination of the 1 day, 3 days, and 28 days strengths. Each group consisted of three samples. Each group of SLM samples was first tested for flexural strength and then tested for compressive strength. For the flexural strength test, the average of one group of three specimens was determined as the flexural strength. The samples which were subjected to the flexural test were also used for the compressive strength test, and the compressed area was 40 mm × 40 mm. The average of six test results was determined as the compressive strength.

## 3. Results and Discussion

### 3.1. Fluidity

The initial fluidity and 20 min fluidity of SLM with 0%, 0.5%, 1.0%, 1.5%, 2.0%, 3.0%, and 4.0% RP were tested, as shown in Table 4.

Table 4 shows that, in comparison to control formulation (R0), the formulations prepared with different dosages of RP showed different initial fluidity and 20 min fluidity. The initial fluidity and 20 min fluidity of SLM changed with the increase in the dosage of RP. The results showed a trend of gradually decreasing first, then increasing, and finally decreasing rapidly. When the dosage was less than 1.5%, the initial fluidity and 20 min fluidity of SLM gradually decreased with the increase in the dosage of RP. Moreover, when the dosage of RP was 1.5%, the fluidity was the lowest. The initial fluidity and 20 min fluidity of SLM with 1.5% RP were 129 mm and 125 mm, which were 9.2% and 8.8% lower than those of R0. This shows that the addition of RP reduced the fluidity of SLM within a certain range.

The fluidity increased gradually as the RP dosage increased from 1.5% to 3.0%. The fluidity of SLM with 3.0% RP was the highest. The initial fluidity and the 20 min fluidity were 143 mm and 139 mm. Compared with the control formulation, the improvements were 0.7% and 1.5%, respectively. The initial fluidity and 20 min fluidity of SLM with 4.0% RP were 9.1% and 10.1% lower than those of SLM with 3.0% RP. This indicated that, when the dosage exceeded a certain range, the fluidity was significantly reduced. Therefore, the dosage of RP should not be too high.

There are two main reasons why the fluidity of mortar changed with the dosage of redispersible powder. Firstly, when the RP is dissolved in water, some of the water needs to be consumed. Moreover, the viscosity of the RP after dissolving increases the cohesion of the slurry, which leads to a decrease in fluidity. On the other hand, due to the high viscosity of RP, the viscosity of SLM increases when RP is added to the mortar. The bubbles introduced in the mixing process and the gas generated in the cement mixing process cannot be discharged. The slurry density on the surface of mortar decreases, the adsorption capacity increases, and the surface area of cement particles increases, which further leads to the increase in slurry viscosity and the decrease in slurry fluidity. Secondly, RP accumulates on the surface of hydrated and unhydrated products, which reduces the internal friction of mortar. In addition, the surface active substances contained in the mortar produce a large number of tiny bubbles, which can be used as tiny balls when they move inside the mortar. Because of the sliding friction between the original cement particles, bubbles play a lubricating role between the particles, the sliding friction resistance in the mortar is greatly reduced, and the fluidity of the mortar increases.

The fluidity of mortar decreased gradually first, then increased, and finally decreased rapidly with the increase in the dosage of RP, due to a superposition of the above two effects. When the dosage was low, the thickening and viscosity of RP were dominant. With the increase in dosage, the fluidity decreased gradually. However, with the increase in dosage, the lubricating effect gradually exceeded that of the viscosity, and the fluidity of mortar gradually increased. However, the phenomenon of agglomeration appeared when too much RP was added, resulting in a rapid decline in fluidity.

### 3.2. Bond Strength

**Figure 3:**
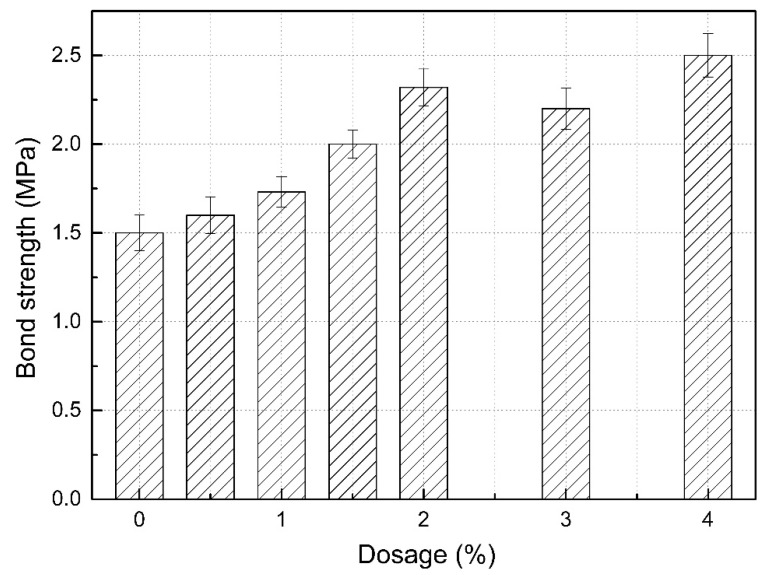
Bond strength of SLM with different dosages of RP.

The bond strength results of SLM prepared with different dosages of RP are shown in Figure 3.

As can be seen from Figure 3, with the increase in the dosage of RP, the bond strength of SLM generally increased gradually. The bond strength of SLM without RP was 1.5 MPa. When the dosage of RP was 4.0%, the bond strength was the largest (2.5 MPa, 66.7% higher than that of SLM without RP).

The reason for the increase in bond strength is that RP is the powder obtained from the spray-drying of a polymer emulsion. The addition of RP promotes the formation of organic polymers and significantly improves the bond strength of mortar by forming films at the mortar interface and in the pores inside. On the other hand, the increase may have been because the flocculating network structure formed by the RP during hydration increased the toughness inside the mortar, thus improving the adhesive performance of the mortar. In this study, no image at the microscopic level was obtained; thus, the above reasons are presumed. However, the study of Qiao et al. [33] could be used to support this presumption, where it was observed that polymers formed a continuous film between the sand and cement hydration products under a scanning electron microscope, which strengthened the bonding between sand and cement slurry, which was an important factor in improving the bonding strength of mortar.

### 3.3. Shrinkage Rate

**Figure 4:**
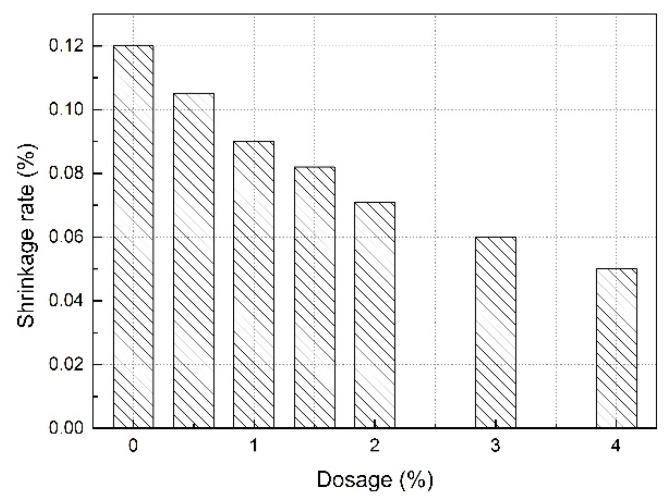
Shrinkage rate of SLM with different dosages of RP.

The shrinkage rate of SLM prepared with different dosages of RP can be seen in Figure 4.

As can be seen from Figure 4, with the increase in the dosage of RP, the shrinkage rate of mortar significantly decreased. The shrinkage rate decreased gradually from 0.12% to 0.05% as the RP dosage increased from 0% to 4.0%. The results show that the RP had an obvious effect on reducing the shrinkage of SLM.

There are two possible reasons for the decrease in shrinkage rate of SLM. Firstly, under the action of polymer active groups, the water consumption required for cement hydration is greatly reduced, and the water utilization rate required for hydration is increased to some extent. After the excess free water evaporates in the environment, the probability of pore retention also decreases. Secondly, the polymer film formed by RP fills the large gap in cement slurry and forms an interwoven membrane network. This kind of spatial network structure also limits the shrinkage of mortar to a certain extent. The studies of Qiao et al. [33] and Shi et al. [34] could be used as support for the above reasons. Under a scanning electron microscope, Qiao et al. and Shi et al. observed that the polymers adhered to the surface of the cement gel or particle and formed a film, filling the pores, thus improving the density of the mortar.

Compared with the samples without RP, when the dosage of RP was 1.0%, 2.0%, 3.0%, and 4.0%, the shrinkage rate decreased by 25%, 41%, 50%, and 58%, respectively. It can be seen that, with the increase in the dosage of RP, the reduction effect of shrinkage rate increased continuously. However, the rate of decrease gradually lessened. For example, the rate of decrease was 25% as the RP dosage increased from 0% to 1.0%, whereas it was 8% as the RP dosage increased from 3.0% to 4.0%. This shows that, with the increase in the dosage of RP, the shrinkage rate of SLM gradually tended to be stable.

### 3.4. Abrasion Resistance

**Figure 5:**
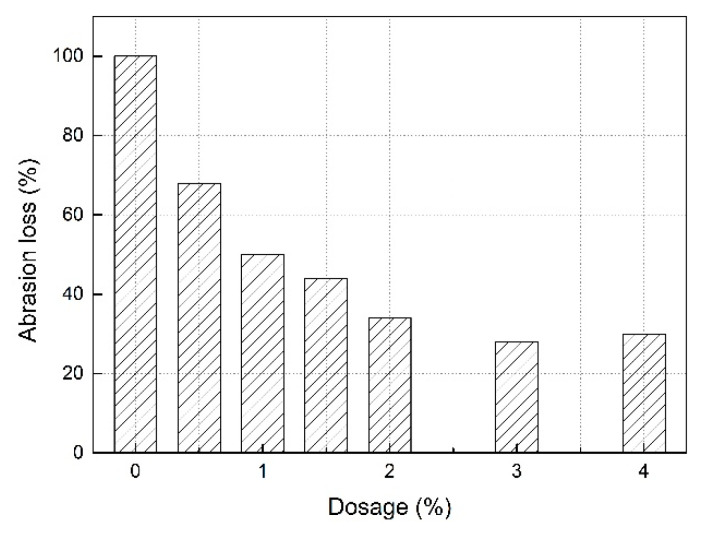
Abrasion loss of SLM with different dosages of RP.

In order to clearly show the effect of RP on the abrasion resistance of SLM, we expressed the abrasion loss as a percentage. The abrasion loss of mortar without RP was recorded as 100%. The abrasion loss results of SLM prepared with different dosages of RP are shown in Figure 5.

As can be seen from Figure 5, with the increase in the dosage of RP, the abrasion loss of SLM first decreased rapidly and then gradually tended to be stable. When the dosage was 3.0%, the abrasion loss of mortar reached the minimum of 28.1%, which was 71.9% lower than that of SLM without RP. The abrasion loss of mortar decreased, which indicates that, within a certain dosage, RP can significantly improve the abrasion resistance of SLM.

The reason for the improvement of the abrasion resistance of SLM is that, after the addition of RP to the SLM, a layer of polymer film is formed on the surface of SLM during the hydration process, resulting in a smooth and hard surface. In the process of abrasion, the polymer film formed by RP retards the development of cracks, reduces the spalling of slurry, and reduces the number of abrasive particles, thus improving the abrasion resistance.

### 3.5. Flexural Strength and Compressive Strength

**Figure 6:**
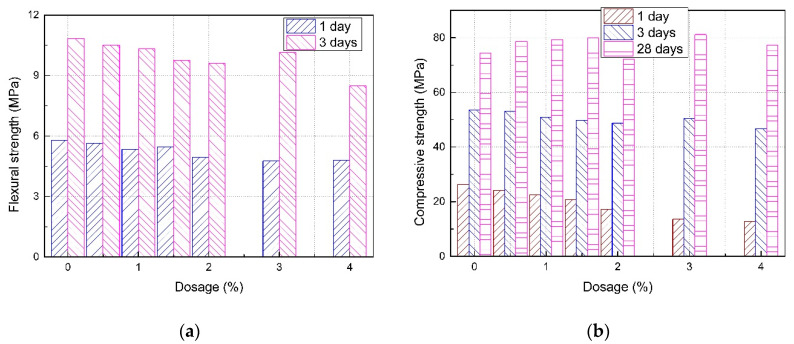
Strength of SLM with different dosages of RP: (**a**) flexural strength; (**b**) compressive strength.

The results of the flexural strength and compressive strength tests of mortar prepared with different dosages of RP are shown in Figure 6. This study tested the 1 day, 3 days, and 28 days flexural strength and compressive strength of SLM. The maximum range of the instrument used in the laboratory to measure the flexural strength was 12.4 MPa, and the flexural strength of the mortar in this experiment after 28 days exceeded this value. Therefore, the specific value of the flexural strength of mortar after 28 days was not measured. Although we did not measure the specific value of the 28 days flexural strength of mortar, it exceeded the specification of 10 MPa [32] and, thus, met the requirements of the standard.

It can be seen from Figure 6a that the 1 day and 3 days flexural strength of SLM basically decreased with an increase in the dosage of RP. The results show that the addition of RP reduced the flexural strength of SLM. At the age of 1 day, the flexural strength of SLM without RP was 5.8 MPa. When the dosages were 3.0% and 4.0%, the flexural strength was about 4.8 MPa, which was 18% lower than that of the mortar without RP. At the age of 3 days, the flexural strength of SLM without RP was 10.85 MPa. When the dosage was 4.0%, the mortar had the lowest flexural strength of 8.5 MPa, which was 22% lower than that of the mortar without RP. Furthermore, with the increase in curing age, the flexural strength of all seven groups of mortar with different dosages of RP increased significantly.

The reason for the decrease in flexural strength of mortar is that, because of the addition of RP, the surface adsorption and film formation of polymer particles inhibit the hydration of cement [35].

It can be observed from Figure 6b that the compressive strength of SLM at different ages showed different trends with the increase in RP. With the increase in RP, the compressive strength of SLM decreased gradually when the age was 1 day; when the age was 3 days, the compressive strength decreased slightly; when the age was 28 days, the compressive strength was basically greater than the compressive strength of SLM without RP. This indicates that the addition of RP reduced the early compressive strength of SLM and improved the later compressive strength.

At the age of 1 day, the compressive strength of SLM without RP was 26.27 MPa. When the dosage was 4.0%, the compressive strength was 12.85 MPa, which was 51% lower than that of the mortar without RP. This indicates that the RP significantly reduced the 1 day compressive strength of mortar. At the age of 3 days, the compressive strength of SLM without RP was 53.61 MPa. When the dosage was 4.0%, the compressive strength was 46.69 MPa, which was 13% lower than that of the mortar without RP. This shows that the RP had little influence on the 3 days compressive strength of mortar. At the age of 28 days, the compressive strength of the mortar after mixing with RP was slightly increased compared with that without RP, except when the dosage was 2.0%. The 28 days compressive strength of mortar without RP was 74.33 MPa. When the dosage of RP was 3.0%, the 28 days compressive strength of mortar was the highest, 81.15 MPa, which was 8.4% higher than that of the mortar without RP. This shows that RP increased the 28 days compressive strength of mortar, but the increase was relatively small.

RP reduced the early compressive strength of mortar, and the 1 day strength reduction was greater than the 3 days reduction. This may be due to the fact that, after the RP is mixed with mortar, on the one hand, the network structure formed by the polymer film and cement hydration products weakens the hydration process of cement particles [35] and reduces the formation of gel of cement hydration products, thus reducing the compressive strength. On the other hand, the surfactant in the RP leads to the introduction of a large number of small bubbles in the mortar, resulting in a large number of small pores in the hardened mortar [36], thus reducing the early strength. The reason for the increase in 28 days compressive strength of mortar may be that, with the formation of hydration products at a later stage and the formation of a flocculent film of RP, the compactness and compressive strength can be improved [33,34].

## 4. Analysis of Reasonable Dosage

The SLM used for ground materials must have good fluidity, strength, stability, cohesiveness, and abrasion resistance to meet the needs of the project. Therefore, in order to determine the reasonable dosage of RP, according to the experimental results, this study took fluidity, compressive strength, flexural strength, stability, cohesiveness, and abrasion resistance as evaluation indices, followed by establishing an evaluation model using the AHP method [37,38,39]. By determining the weighted value of each index to evaluate the performance of mortar, the reasonable dosage of RP was analyzed.

**Figure 7:**
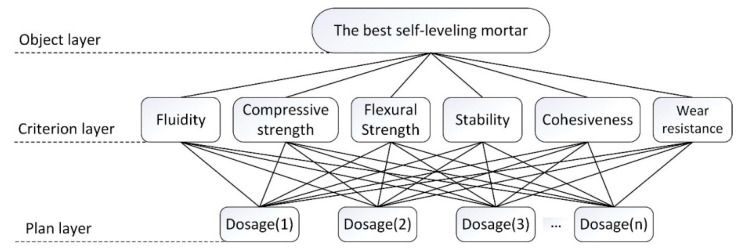
Hierarchy structure model for performance evaluation of SLM based on experimental tests.

The hierarchical structure model of performance evaluation for SLM is shown in Figure 7. The plan layer denotes the dosage of RP, i.e., dosage (1), dosage (2), dosage (3), … dosage (*n*).

According to the priority and importance of the performance evaluation indices, on the basis of the pairwise comparison of indices, the judgment matrix (A) was generated using Equation (4) according to the point scale (Table 5) of AHP and combined with engineering experience, as shown in Table 6. Each entry a_ij_ in the matrix was built by comparing the row element A_i_ with the column element A_j_ [40].

A = (a_ij_)_k×k_ (i, j = 1, 2, 3, …, k), a_ij_ = 1/a_ji_ (4)

A_1_, A_2_, A_3_, A_4_, A_5_, and A_6_ in the judgment matrix were used to represent the fluidity, compressive strength, flexural strength, stability, cohesiveness, and abrasion resistance of SLM. For the performance evaluation of mortar, because the evaluation indices were not independent variables and their priority was not absolute, it was difficult to achieve an accurate comparison among indices; thus, the judgment matrix was not unique.

To calculate the weighted value according to the judgment matrix, firstly, the maximum eigenvalue and corresponding eigenvector of matrix A were obtained. The eigenvector was normalized to the weighted value of each index. The maximum eigenvalue *λ*_max_ of matrix A was 6.16. The corresponding eigenvector W = (0.422, 0.196, 0.196, 0.089, 0.058, 0.039) was calculated through the AHP. Because the value of judgment matrix was given artificially and had subjectivity, the reliability of the judgment matrix was verified using a consistency check. The consistency index (*CI*) was used to measure the consistency among pairwise comparisons according to the following equation:

(5) $$CI=\frac{\lambda_{\max}-n}{n-1}$$

where *λ*_max_ is the eigenvalue and *n* is the number of indicators in the pairwise comparison matrix. Therefore, according to Equation (5), *CI* was 0.032. The consistency ratio *CR* was utilized to judge the consistency of the matrix according to Equation (6).

(6) CR=CI/RI

where *RI* is the random index, given in Table 7 according to Saaty [41]. Generally, the upper limit of the satisfactory range of *CR* is 0.1.

According to Equations (5) and (6) and Table 7, the following values were obtained: *CI* = (6.16–6)/(6–1) = 0.032; *CR* = *CI*/*RI* = 0.032/1.24 = 0.026 < 0.1. Therefore, the value of W obtained above was acceptable; that is, the calculated relative importance was acceptable. Hence, the weighted values of fluidity, compressive strength, flexural strength, stability, cohesiveness, and wear resistance of mortar were 0.422, 0.196, 0.196, 0.089, 0.058, and 0.039 respectively. The sum of the weighted value of these six indices was 1, which means that the performance of the mortar was determined by these six indices.

The experimental results of performance indices of mortar with different dosages of RP are shown in Table 8. In order to facilitate comparisons of the comprehensive evaluation indices, some original experimental data were simply processed, as shown in Table 8. The compressive strength and flexural strength were expressed as 1 day test results; stability was expressed by shrinkage rate according to the following equation:

(7) $$S_{i}=\varepsilon_{0}-\varepsilon_{i},\;i=\;0,\;0.5,\;1,\;1.5,\;2,\;3,\;4$$

where $\varepsilon_{i}$ refers to the shrinkage rate of SLM when the dosage of RP is *i*%. Cohesiveness was expressed by bond strength. Abrasion resistance was expressed by abrasion loss according to the following equation:

(8) $$W_{i}=F_{0}-F_{i},\;i=\;0,\;0.5,\;1,\;1.5,\;2,\;3,\;4$$

where *F_i_* refers to the abrasion loss of SLM when the dosage of RP is *i*%.

The data listed in Table 8 represent reference values for evaluating the performance of mortar and, thus, should not be neglected. If the dosage of RP was determined only by fluidity, the reasonable dosage would be 3.0%. If only based on the compressive strength, the compressive strength of SLM without RP was the highest. If only in terms of cohesiveness, the reasonable dosage of RP was 4.0%. Obviously, these evaluation results are contradictory and inconsistent. In order to evaluate the performance of mortar more reasonably and determine the reasonable dosage of RP, it is a better choice to consider the weighted value of each index.

The experimental data of six performance evaluation indices were processed in dimensionless form using Equation (9), and then the weighted average value of each evaluation index was calculated according to Equation (10), whereby the weighted average value was regarded as the comprehensive index of mortar performance evaluation, as shown in Table 9.

(9) $$m_{i}=\left|p_{i}/\Sigma p_{i}\right|\times 100$$

(10) $$M=\Sigma m_{i}\cdot \eta_{i}$$

where *P_i_* (*i* = 1, 2, 3, 4, 5, 6) is the performance evaluation index, *m_i_* is the dimensionless value of the performance evaluation index, *M* is the comprehensive index of performance evaluation, and *η_i_* is the weighted value of the performance evaluation index. A higher value of the comprehensive index generally denotes the better performance of mortar. Thus, according to the comprehensive indices of SLM in Table 9, the reasonable dosage of RP was found to be 3.0%.

## 5. Conclusions

The influence of RP on the properties of ternary composite SLM were investigated from the aspects of fluidity, bond strength, shrinkage rate, abrasion resistance, flexural strength, and compressive strength. The weighted value of each index was calculated using the AHP. The weighted value and weighted sum of each evaluation index were used to calculate the comprehensive evaluation index of mortar performance, and the reasonable dosage of RP was determined. The following conclusions can be drawn from the research:

1. The effects of RP on fluidity did not show a general trend but changed with RP dosage. From 0% to 1.5% RP, the fluidity of mortar decreased gradually. From 1.5% to 3%, the fluidity of mortar increased gradually. The fluidity of SLM with 3.0% RP was the highest. The initial fluidity and 20 min fluidity of SLM with 3.0% RP were 0.7% and 1.5% higher than those of SLM without RP. The addition of RP showed a small improvement in the fluidity of SLM.
2. The addition of RP significantly improved the bond strength and abrasion resistance of SLM. When the dosage of RP was 4.0%, the bond strength of SLM was 66.7% higher than that without RP. The SLM with 3.0% RP obtained the lowest abrasion loss of 28.1%, which was 71.9% lower than that of SLM without RP. RP could also reduce the shrinkage of SLM, thereby improving the crack resistance of SLM. Upon raising the dosage of RP from 0% to 4.0%, the shrinkage rate decreased from 0.12% to 0.05%.
3. RP had different effects on the flexural strength and compressive strength of SLM. RP reduced the flexural strength of SLM, whereby the 1 day and 3 days flexural strengths of SLM with 4.0% RP were 17% and 22% lower than those of samples without RP. In terms of the compressive strength, RP reduced the early strength and increased the later strength. The 1 day and 3 days compressive strengths of mortar with 4.0% RP were 51% and 13% lower than those of the mortar without RP. The 28 days compressive strength of mortar with 3.0% RP was 8.4% higher than that of the mortar without RP.
4. AHP was used to establish a hierarchical model of RP dosage and SLM performance to determine the reasonable dosage of RP. According to the AHP, the reasonable dosage of RP for the SLM of a ternary cementitious system (OPC–SAC–CS) was found to be 3.0%.

## Figures and Tables

**Table 1:** Chemical compositions of cements used in this study (wt.%).

Materials	CaO	SiO_2_	Al_2_O_3_	Fe_2_O_3_	SO_3_	MgO	TiO_2_	Loss
OPC	64.95	18.31	4.21	2.95	4.22	0.64	0.23	3.21
SAC	39.25	12.28	37.12	2.36	9.52	-	-	-

**Table 2:** Performance parameters of redispersible powder.

Appearance	Bulk Density (g/L)	Ash Content (1000 °C)	pH	Minimum Film Forming Temperature (°C)	Residual Moisture
White power	400–640	(9–12)%	7.0–8.5	+3	≤1.0%

**Table 3:** Composition of SLM mix formulations (kg/m^3^). CS, calcium sulfate; GCC, ground calcium carbonate.

No.	OPC	SAC	CS	Aggregates	FA	GCC	Admixtures 1	RP
R0	417.6	24	9.6	427	27.1	60	33.4	0
R0.5	417.6	24	9.6	427	27.1	60	33.4	4.8
R1	417.6	24	9.6	427	27.1	60	33.4	9.6
R1.5	417.6	24	9.6	427	27.1	60	33.4	14.5
R2	417.6	24	9.6	427	27.1	60	33.4	19.3
R3	417.6	24	9.6	427	27.1	60	33.4	29.0
R4	417.6	24	9.6	427	27.1	60	33.4	38.6

**Table 4:** Effect of RP on the fluidity of SLM.

No.	Dosage (%)	Fluidity (mm)	
		Initial	20 min
R0	0	142	137
R0.5	0.5	135	133
R1	1.0	133	129
R1.5	1.5	129	125
R2	2.0	132	129
R3	3.0	143	139
R4	4.0	130	125

**Table 5:** Saaty’s 1–9 point scale.

Point Scale	Definition
1	Equal importance of both element
3	Moderate importance of one over another
5	Strong importance
7	Very strong importance
9	Absolute importance
2, 4, 6, 8	Intermediate values between the two adjacent scale values

**Table 6:** Judgment matrix.

Item	A_1_	A_2_	A_3_	A_4_	A_5_	A_6_
A_1_	1	3	3	5	6	7
A_2_	1/3	1	1	3	4	5
A_3_	1/3	1	1	3	4	5
A_4_	1/5	1/3	1/3	1	2	3
A_5_	1/6	1/4	1/4	1/2	1	2
A_6_	1/7	1/5	1/5	1/3	1/2	1

**Table 7:** Random index (RI) values for different scales.

Scale	1	2	3	4	5	6	7	8	9
RI	0.00	0.00	0.58	0.90	1.12	1.24	1.32	1.41	1.45

**Table 8:** Parameters for performance evaluation of SLM.

No.	Fluidity	Compressive Strength	Flexural Strength	Stability	Cohesiveness	Abrasion Resistance
R0	142	26.27	5.8	0	1.5	0
R0.5	135	24.17	5.65	0.015	1.6	0.50
R1	133	22.53	5.34	0.030	1.7	0.80
R1.5	129	20.76	5.46	0.038	2.0	0.90
R2	132	17.22	4.95	0.049	2.3	1.05
R3	143	13.57	4.76	0.060	2.2	1.15
R4	130	12.85	4.8	0.070	2.5	1.12

**Table 9:** Dimensionless values of parameters for performance evaluation and the comprehensive index.

No.	Fluidity	Compressive Strength	Flexural Strength	Stability	Cohesiveness	Abrasion Resistance	Comprehensive Index
R0	15.04	19.12	15.78	0	10.83	0	13.82
R0.5	14.30	17.59	15.37	5.73	11.55	9.06	14.03
R1	14.09	16.40	14.53	11.45	12.49	14.49	14.32
R1.5	13.67	15.11	14.85	14.50	14.44	16.31	14.41
R2	13.98	12.54	13.46	18.70	16.75	19.02	14.37
R3	15.15	9.88	12.95	22.90	15.89	20.83	14.64
R4	13.77	9.36	13.06	26.72	18.05	20.29	14.42
